# The Diagnosis of Diseases of the Brain, Spinal Cord, Nerves, and Their Appendages
*"The Diagnosis of Diseases of the Brain, Spinal Cord, Nerves, and their Appendages. By J. Russell Reynolds, M.D., &c. London. 1855."


**Published:** 1856-01-01

**Authors:** 


### REVIEWS.

### THE DIAGNOSIS OF DISEASES OF THE BRAIN, SPINAL CORD, NERVES, AND THEIR APPENDAGES.*

To supply the practitioner and student with a concise manual of Diagnosis of Nervous Diseases, is the object Dr. Reynolds has in view in this work.

* "The Diagnosis of Diseases of the Brain, Spinal Cord, Nerves, and their Appendages. By J. Russell Reynolds, M.D., &c. London. 1855."

In the present state of our knowledge, when the physiology of the nervous system is involved in so much obscurity, any attempt to classify its diseases on a purely pathological basis must result in failure. Dr. Reynolds contents himself at present with symptoms and physical signs, upon which he founds his classification—open it is true to many grave objections, as we shall shortly see—but presenting one decided advantage, namely, that it involves no theory.

In the first part of his book, the author considers in a general manner the objects and elements of diagnosis, and the classification of nervous diseases. The second part is devoted to diseases of the brain; the third, to diseases of the spinal cord; and the fourth, to those of the nerves and their appendages.

The following analysis will supply the reader with an idea of Dr. Reynolds' views.

The first chapter treats of the objects of diagnosis, which are three in number, "first, the locality; second, the nature; and third, the anatomical conditions of the lesion." The symptoms by which we are guided to the locality are "extrinsic" or constitutional, and "intrinsic," or local. As to the nature of the affection, it may be "acute" or "chronic."

The anatomical conditions may be "simple functional derangement,"—which the author supposes in the case of "epilepsy, chorea, hysteria, neuralgia," &c.; or it may be a "physical change, as in organic diseases."

The second chapter considers the elements of diagnosis; by which "is intended the symptoms of disease, which furnish means by which diagnosis may be established."

"The intrinsic, or proper nervous symptoms, are essentially modifications of the manner in which the organs of the nervous system perform their functions;" they may be "mental, or connected simply with motility and sensibility."

The mental phenomena referrible to volition, may be in relation to ideas, as when there is "modification of the power of attention," "modified power of apprehension," "changes in the faculty of recollection," or "modifications in the power of directing thought."

Volition, in its relation to emotion, may be affected by "diminished control of emotion," or by "diminished contrast of expression;" both prominent features in various forms of insanity. "The two need not coexist; the former is an internal change, sometimes to be discovered only by diligent search, and by gaining the 'confidence' of the patient; the latter betrays itself at once in his tone, manner, and gesticulation." Diseased volition in relation to sensation may show itself by the "morbid quickness of perception," as instanced by "the hypochondriac, who not only exaggerates all his sensations, and with unhealthy rapidity interprets them to his own discomfort, but can create them in accordance with his preconceived ideas;" or by "the maniac, who can with marvellous quickness of intuition adapt everything that the individual hears, feels, or sees, into some confirmative evidence of his own delusion."

The opposite condition of this, is when there is diminished perceptive power, which may be the case in delirium, or in that state where the patient "lies perfectly motionless, cannot be made to utter a sound, and makes no attempt to do so spontaneously." The author takes exception to the phrase, "loss of consciousness," as applied to this condition, "because," says he, "it is merely an assumption that such loss exists—an assumption which the after-evidence of many cases has proved to be incorrect." He proposes instead, "loss of perception," as "conveying what exists in fact."

We doubt if this brings us much nearer the truth. A case occurs to us, in which a man lay for months in this condition, apparently having lost alike consciousness and perception, and who on recovering, showed that he had not only perceived what was going on around him—even to the motions of the spiders in his room—but had formed his likes and dislikes to those attending on him in proportion as they had been kind or unkind in their treatment. In fact, it is one of the secrets of the moral treatment of the disease, that in general the patients not only perceive and appreciate, but even remember acts of kindness or cruelty, often when they do not appear to do so.

"Volition in reference to motility," may be affected in its "power of occasioning movement," either by excess as in the maniac, or by deficiency as in hysteric paralysis, so common in the female sex, termed sofa disease.

There may be a defect in the "power of directing movements," or in the "control of involuntary movement." The mental phenomena referrible to "ideation," or "modification in the processes of thought," may have relation to "external impressions," either by abstraction from their influence as in mental "absence," or by perverted notions of their nature or relation.

Again, ideation in relation to internal sensations, may be affected as in hypochondriasis, &c.; or it may be as "an independent process," either by "loss of power to appreciate the logical sequence of events," by the "sequence of ideas" being "rapid, but accidental," by "the absence of all discoverable sequence," by the "loss of memory in its severer forms," by "positively exaggerated ideation," which is seen in some forms of delirium, or by "perverted ideation, or the existence of fixed delusions," as in insanity. "Ideation in relation to motility," is implicated in "the hypochondriac, the hysteric, and the choreic patient," or in electro-biologic subjects, where muscular movements are effected in opposition to the will.

The mental "symptoms referrible to emotion," consist in morbid exhibitions of pleasure, displeasure, joy, sorrow, &c., either by their exaggeration, perversion, or diminution.

The intrinsic symptoms which are not mental, are referrible to sensation or sensibility, or to motility. Sensibility may be affected in one of these ways, it may be increased, diminished, or modified, so as to produce false sensations.

The phenomena of motility "resolve themselves into muscular contraction or its absence."

The author classifies them thus:—

"*a.* Modified relation of motility to volition.

" *b*. Motility as induced by ideation.
" *c*. Disordered relation of emotion and motility.
" *d*. Motility in relation to sensation.
" *e*. Motility in relation to reflection, or a sensual impression.
" *f*. Motility in relation to centric irritation.
" *g*. Motility in relation to electric stimulation.
" *h*. Proper motility of the muscles."

We omit any notice of the extrinsic symptoms, as there is nothing peculiar in the author's arrangement of them.

In Chapter III. Dr. Reynolds explains the classification he has adopted, and his reasons for doing so, which are simply that he considers it the most convenient for his purpose—namely, diagnosis.

The three objects of diagnosis, as stated above, are " locality, nature, and lesion;" and these are his guides in the following classification:—

I. Diseases of the encephalon.
  A. Acute.
    1. Febrile, or inflammatory.
    2. Non-febrile.
      *a*. Apoplectic diseases.
      *b*. Diseases marked by delirium.
      *c*. Convulsive diseases.
      *d*. Diseases marked by pain.
  B. Chronic diseases.
    1. Marked by increased activity.
      *a*. Ideation, its characteristic being hallucination, &c.
      *b*. Sensation, its characteristic being pain.
      *c*. Motility, its characteristic being spasm.
    2. Marked by diminished activity.
    3. Marked by the combination of increased and diminished activity.
II. Diseases of the spinal column and cord.
  A. Acute.
  B. Chronic.
III. Diseases of the nerves.
  A. Structural, or organic.
    1. Neuritis.
    2. Tumor.
  B. Functional, or dynamic.
    1. Neuralgia, and spasm.
    2. Anæsthesia, and paralysis.

Chapter IV. treats of the diagnosis of locality generally. We have to distinguish between " diseases of the nervous system itself," and " the nervous complication of other diseases;" also " affections of the brain, spinal cord, and nerves from one another;" and between " meningeal and central lesions."

In diseases of the nervous system—

" 1. Prodromata are of intrinsic character, or absent;
" 2. Signs of distinct general disease are undiscoverable;
" 3. The intrinsic symptoms precede such general or extrinsic symptoms as may be present, and are of greater relative intensity than the latter will account for."

When the nervous symptoms are but complications of some general or some extrinsic disease—

"1. The prodromata are highly marked, and consist of extrinsic symptoms;

"2. The signs of general (or extraneous) disease are discoverable;

"3. The extrinsic symptoms have not only preceded the intrinsic, but the latter bear a definite and direct proportion to the former; and the extrinsic derangements are more highly marked than those which the supposed nervous conditions could induce."

The points to be attended to in distinguishing disease of the brain, spinal cord, and nerves from each other, are—

"1. When perception, ideation, volition, and special sensations are affected; and motor and general sensory changes exhibit a unilateral distribution, the brain is commonly the seat of the disease.

"2. When the mental functions are unchanged, and motility and general sensibility are affected bilaterally, we infer the spinal cord to be the locality of the lesion.

"3. When the relations between motility, volition, and reflection are lost, the mental functions being unchanged, and when the motor and sensory disturbances are purely local, we refer the disease to some of the nervous trunks."

The diagnosis between centric and excentric diseases of the nervous system is guided by the following general characters. At the outset, or at a very early stage in the development of a centric disease, there is "loss of some one or more of the proper nervous functions, such as by paralysis, anæsthesia, loss of memory, &c."

Whereas, in "meningeal diseases there is extremely severe excitement or exaggeration of function, such as furious delirium, anæsthesia, convulsions, and well marked epiphemena, pain, tenderness, &c."

The second part of Dr. Reynolds' work is occupied by the classification of diseases of the brain and their symptoms. In Chapter V. he divides acute and chronic diseases into various groups; the former into "febrile, apoplectic, delirious, and convulsive;" the latter into those marked by excessive activity of some functions; those characterized by diminution, and those presenting, in combination, the features of the latter two."

This classification is avowedly a faulty one; it not only brings together diseases widely different in their nature, but separates others which, if not identical in their pathology, are closely allied. The author himself is not blind to those objections; he says—"Although, therefore, it will be found that softening of the brain (for example) occurs in the apoplectic, delirious, convulsive, and quasi-febrile form, I prefer considering that peculiar condition of the brain in conjunction with its several groups of symptoms as representing four different conditions of the disease, rather than looking upon them as variable phenomenal phases of the same malady."

The sixth chapter is devoted to the "Differential diagnosis of acute febrile diseases affecting the brain."

These are as follows:—

"I. Meningitis, or inflammation of the pia mater, distinguishing—
  A. Simple—*i.e.*, non-diathetic, or primary, when affecting—
    1. The convexity of the hemispheres.
    2. The base of the brain.
  B. Tuberculous, accompanying deposit in the pia mater.
  C. Rheumatic, or meningeal rheumatism.

"II. Inflammation of the dura mater.

"III. Cerebritis, commonly meningo-cerebritis.
  A. General, and then always meningo-cerebritis.
  B. Partial, or limited (red softening).

"IV. Continued fever (typhoid and typhus) with cerebral complication.

"V. Gastric remittent fever of children.

"VI. Simple hyperæmia, or 'determination of blood.'

"VII. Delirium tremens, in its febrile form.

"VIII. Mania, with marked febrile symptoms."

"Meningitis of the base" is seldom to be distinguished from "meningitis of the convexity of the hemispheres;" but when we see "intelligence being preserved for a time (without delirium), and coma, or somnolence, occurring very early in the disease," the base is probably its seat.

"Tubercular meningitis" is presented under two forms, the first occurring in the child, the second in the adult. "Mutism is not uncommon" in this disease. The author quotes Dr. Wahle as having drawn attention to this symptom. The diagnosis of rheumatic meningitis is based upon the facts of—

1. Rheumatic fever being present in a—
2. Weak or exhausted subject; and the sudden occurrence of—
3. Delirium, of marked, furious character,
4. Cephalalgia, and
5. Spasmodic movements, partial or general, followed by a—
6. Comatose condition, with paralysis.

"Inflammation of the dura mater" is generally the result of the suppression of a chronic discharge from the ear.

Unless complicated with inflammation of the pia mater, the furious delirium of meningitis is supplanted by oppression, drowsiness, and coma.

"Cerebritis" may be "general" or "local;" there is "confused thought," and general obscurity of the intellectual faculties—absence of "excitement;" and in the partial form or "red softening," "loss of power," with "tingling and numbness in one limb, or side."

"Hyperæmia cerebri," or cerebral congestion, "resembles very closely," as, indeed, it probably is, the first stage of meningitis, from which it differs only in its negative characters.

The febrile form of "acute mania" is so easily known from meningitis and cerebritis by the mental phenomena, that it is unnecessary to mention its symptoms.

The other acute febrile diseases affecting the functions of the brain are continued fever, "gastric remittent fever," and the febrile form of "delirium tremens."

In the two former the head symptoms are accidental, and in the latter the previous history, as well as the characteristic form of delirium, serve to distinguish it from allied diseases.

The seventh chapter is devoted to "apoplectic diseases;" there are—

"I. Congestion of the brain, or 'coup de sang.'

"II. Hæmorrhage, extravasation of blood ('apoplexy' proper).

"A. Hæmorrhage into the substance of the hemisphere.

"B. Ventricular hæmorrhage.

"C. Arachnoid hæmorrhage.

"III. Serous effusion in large quantity. ('Serous apoplexy.')

"IV. Local cerebritis, or 'softening of the brain.'

"V. Tumour of the brain, or meninges.

"VI. Tubercular meningitis.

"VII. Urinæmia and diathetic states.

"VIII. Anæmia, morbus cordis, vascular obstruction."

The phenomena of apoplexy are too well known to require specification. Dr. Reynolds says, "The essential nature of apoplexy is the occurrence of some static or dynamic change which, *pro tanto*, severs volition and perception (the brain functions) from motion and sensation."

"As congestion frequently accompanies or precedes all apoplectic diseases, its symptoms are often present as their prodromata. Where congestion, however, forms the whole anatomic basis of developed apoplexy, they are more marked in intensity, and have commonly existed for a longer period." Hæmorrhagic apoplexy is characterized by the suddenness of its invasion. "The patient, as a rule, if standing, falls instantly, as if knocked down." The accompanying paralysis is generally hemiplegic if the hæmorrhage be into the substance of the hemispheres. If it be into the ventricle, "the most frequent combination of symptoms" is profound coma, with general paralysis and rigidity. In arachnoid hæmorrhage the symptoms are more slowly developed, and "rarely simultaneously."

"There is no certainty in the diagnosis of "serous apoplexy."

"The clinical history" of "acute red softening" "closely resembles that of cerebral hæmorrhage" . . . . "in some case the differentiation is impossible." Apoplexy may occur in the course of the growth of a "tumour of the brain," or in the progress of "tubercular meningitis;" it may also be the result, or at least the accompaniment, of various poisoned states of the blood, as in Bright's disease, jaundice, or diabetes.

"Morbus cordis," anæmia, and vascular obstructions are the remaining causes of apoplexy.

The diseases (Chap. VIII.) marked by delirium, unaccompanied by fever, are—

"I. Hyperæmia of the brain and meningitis.

"II. Partial cerebritis, or red softening.

"III. Delirium tremens.

"IV. Extrinsic diseases, including urinæmia, icterus, diabetes."

The first of these is marked by "the simplicity of the delirium—*i. e.* its freedom from complication with other intrinsic nervous symptoms."

In "acute softening," the "delirium is mild and inoffensive," and in the intervals of delirium there is distinct mental weakness, loss of memory, confusion of ideas, &c.

Delirium tremens, on the other hand, is "of a fearful, wandering, but tractable type, with delusions; a peculiar tremor, wakefulness, a non-febrile state, with clammy, cool skin, and disordered, offensive secretions."

In "diathetic diseases," "the delirium is commonly mild, and 'low muttering' in its character, attended by subsultus tendinum, or chronic spasms."

Convulsions (Chap. IX.) may have a "centric" or an "eccentric" origin. The latter are—

"I. Blood diseases, or toxæmiæ.

"1. Introduced poisons, including the acute specific diseases, the exanthemata, mineral poisons, &c.

"2. Retained poisons, or excreta, such as urinæmia, icterus, rheumatism (?), &c.

"II. Eccentric irritations (not toxæmiæ).

"1. Gastro-intestinal dentition, dyspepsia, worms, constipation, &c.

"2. Bronchio-pulmonary. Laryngismus, pertussis, &c.

"3. Genito-urinary. Morbid uterine conditions, calculoid affections, &c."

The "convulsive diseases of intrinsic origin (centric)" are—

"III. Idiopathic, without assignable static cause.

"IV. Congestion of the brain, and meningitis.

"V. Softening of the brain (local acute cerebritis).

"VI. Tubercular meningitis.

"VII. Tubercle and tumour of the brain.

"VIII. Cerebral hæmorrhage.

"IX. Cerebral hypertrophy.

"X. Acute chorea.

Cephalalgia as an acute symptom may be of extrinsic or intrinsic origin; under the former we have it, 1, in the acute specific diseases; 2, rheumatic cephalalgia; 3, sympathetic headache. Where of intrinsic origin it may be, 1, congestive; 2, inflammatory; 3, connected with organic diseases; 4, neuralgic.

Of chronic diseases of the brain, the first, treated of in Chapter XII., are those "characterized by exalted activity."

"A. Excessive ideation.

"I. Hypochondriasis.

"II. Tarantism.

"B. Excessive sensation.

"III. Hemicrania, or hyperalgesia cerebri.

"IV. Hallucinations.

"V. Illusions (vertigo of sensation, &c.).

"C. Excessive motility.

"VI. Vertigo of motion (rotatory movements).

"VII. Co-ordinated spasm (muscular tic).

"VIII. Chorea.

"IX. Tremor (paralysis agitans)."

The diagnosis of hypochondriasis from melancholia, says the author, "is based upon the hypochondriac's constant self-regard, and the habitual reference of his delusions to the corporeal sphere."

"The predominance of motor disturbance" in hysteria "will generally serve to distinguish" it from hypochondriasis.

By "hallucinations," the author means those which are unconnected with insanity; so that the subject of them, "although his phantasms may have the appearance of reality, does not believe in their objective existence."

A somewhat similar distinction should be drawn between the illusions of the sane and insane. Muscæ and tinnitus aurium are illusions common to every one, and the result of a real impression on the sensory nerve; but where the muscæ, on the one hand, are firmly believed to be furies or devils, or the ringing in the ears, on the other, is transformed into "voices," then the mind is insane.

The same thing holds good in reference to optical illusions, as spectra; the sane mind can by experiment convince itself of their real nature, whereas no process of reasoning will ever unseat the delusive impressions of the insane.

"The most important chronic diseases of the brain, and nervous system generally, present a combination of exaggerated activity in some portions and diminished function in others."

Those so characterized are as follow:—

"I. Hysteria, and allied affections, catalepsy, &c.

"II. Epilepsy, 'le haut' and 'le petit mal.'

"III. Tumour of the meninges, cerebrum, and cerebellum.

"1. Carcinomatous, } sometimes separable.

"2. Tuberculous, }

"3. Aneurismal, fibroid, hydatid, &c., not separable.

"IV. Chronic meningitis.

"V. Chronic softening.

"VI. Induration of the brain.

"1. In the adult (from epilepsy, lead poisoning, &c.)

"2. In the child (hypertrophy of brain).

"VII. Chronic hydrocephalus.

"VIII. Urinæmia."

There is nothing pathognomonic in the symptoms of specific tumours. The tuberculous and carcinomatous are inferred by the presence of the cachexia; the aneurismal by the existence of arterial disease elsewhere; while the other varieties may be guessed at from the discovery of similar growths in other parts of the body.

As indications of the "special locality" of a tumour, the following are valuable. "Pain is most commonly situated on the same side as that in which the tumour exists." "Motor phenomena (both spasmodic and paralytic) are observed almost invariably on the opposite side."

"Convulsions are most frequent in tumours of the cerebellum."

"Amaurosis, on the other hand, is most common in tumours of the anterior cerebral lobes."

"The implication of the special senses generally (but not exclusively) indicates a location near the base."

A suggestion of Romberg's, confirmed by one case observed by Dr. Reynolds, will form a valuable means of diagnosis, if more extended observation proves it to be trustworthy; namely, that when the tumour is situated on the upper surface of the encephalon, a "forced expiration increases the pain;" whereas when affecting the base, "this effect is produced only by inspiration."

"Paraplegia rarely occurs from encephalic tumour, unless the cerebellum is its seat."

"When softening has observed a chronic course throughout, its most difficult differentiation is from tumour and meningitis. The three may, however, be distinguished in many cases by the following characters.

"A. Tumour,—intense, locally limited, paroxysmal pain; anæsthesia of special senses; local paralyses; epileptoid convulsions without paralyses; unimpaired intelligence; coma at close of life.

"B. Chronic meningitis,—pain, not very severe, not limited; mental and emotional excitement; disorderly spasms and paralyses; with frequent, but irregular accessions of fever.

"C. Chronic softening,—oppressive, not intense pain; with gradual failure of intelligence, motility, and sensibility."

The nervous symptoms of urinæmia may resemble those of these three affections, but then "the pain is rarely acute; there is drowsiness, or a peculiar coma and stertor, and the extrinsic symptoms furnish the means by which a diagnosis may be established."

The third part of the book is devoted to diseases of the spinal cord.

With regard to the special locality—the cervical, dorsal, or lumbar regions may be affected.

When the lumbar or lower dorsal portions of the cord are the seats of disease, the "lower limbs are alone implicated." "The bladder and rectum are paralyzed." If the upper dorsal region be affected, "respiration is impeded;" "unless the lesion extends above the second dorsal vertebra, the upper limbs retain their function." "Affections of the cord opposite the first dorsal, or the last two cervical vertebræ, implicate the movements of the arms."

"If the disease extends no higher than the sixth cervical, the arms retain their movements at the shoulders;" if above the sixth or fifth, "and the phrenic nerve is implicated, the dyspnœa is most urgent."

"If the lesion exists higher than the fourth or third vertebra, death is extremely rapid, owing to asphyxia from paralysis of the respiratory muscles."

"The locality of disease may be discovered by the existence of spontaneous pain, or tenderness, at a particular point of the vertebral column; and the latter may be estimated by pressure, concussion of the spinous processes, or the application of heat (by means of a sponge or cloth wrung out of hot water)."

"Where motility is at first exclusively affected, the anterior and antero-lateral columns are most probably diseased; and *vice versâ*, when sensibility is primarily deranged, the probability is, that the posterior, or postero-lateral columns are principally affected."

"Acute diseases of the spinal cord and its meninges" are as follows:—

"I. Plethora spinalis, or congestion.
"II. Meningitis.
"III. Myelitis (acute softening).
"IV. Meningo-myelitis.
"V. Tetanus (idiopathic).
"VI. Hydrophobia.
"VII. Hæmorrhage, meningeal and spinal.
"1. Into the spinal cord.
"2. Into the tuber annulare.
"VIII. Concussion of the cord."

Spinal meningitis is ushered in by "highly marked fever," and is accompanied by "pain referred to the spine, at first slight, but rapidly increasing in severity, and becoming almost intolerably violent." Tonic spasm is the chief motorial symptom.

Myelitis, on the other hand, is denoted by "peripheral pain, or anæsthesia, and paralysis;" it is "commonly hyper-acute, and terminates in a few days; but if this is not the case, sloughing of the integuments occurs, and hastens the prostration of the patient to a final issue."

Meningo-myelitis "is more common than either of its elements in an isolated form."

The chronic diseases of the spinal cord are—

"I. Chronic myelitis (or softening).
"II. Chronic meningitis.
"III. Induration and hypertrophy.
"IV. Tumours.
"1. Diathetic—*e.g.*, tubercle, carcinoma.
"2. Non-diathetic—*e.g.*, hydatids.
"V. Idiopathic paraplegia (dynamic)."

In the fourth and last part of this work, Dr. Reynolds considers the diseases of the nerves.

The diagnosis of the particular nerve affected "can be arrived at only by a knowledge of the anatomical distribution," "and physiological functions of each division."

The functions of a nerve may be modified by—

"I. Excessive activity.
"A. Of sensation or sensibility.
"B. Of motility.
"II. Diminished activity, or complete loss of function.
"A. Of sensation, or rather of impressibility.
"B. Of motility."

The special diseases of the nerves are thus arranged:—

"I. Neuritis (inflammation of the nerve trunks).

"II. Tumours; of two kinds.

"*a.* Painful subcutaneous tubercle.

"*b.* Neuroma (of various kinds).

"B. Inorganic or functional.

"III. Neuralgiæ, considering specially,—

"*a.* Facial. Neuralgia of the fifth nerve.

"*b.* Ischiatic. Sciatica.

"*c.* Dorso-intercostal.

"IV. Hypercineses, or spasms, considering specially,—

"*a.* Facial-spasmodic tic.

"*b.* Oculo-motor. Strabismus.

"*c.* Laryngeal. Laryngismus stridulus.

"V. Anæsthesiæ, especially of the fifth nerve.

"VI. Acineses, or paralyses, and especially that of the facial nerve (portio dura of the seventh)."

---

